# Supplementary figures and images for: Development and evaluation of a deep learning model for protein–ligand binding affinity prediction
Source: Bioinformatics. 2018 May 10;34(21):3666–74. doi: 10.1093/bioinformatics/bty374 (PMC6198856; doi:10.1093/bioinformatics/bty374)

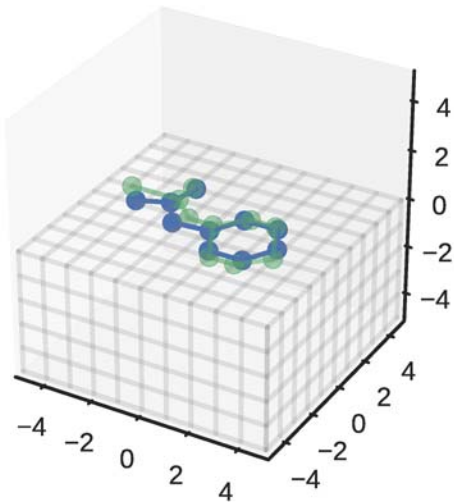

Supplement: Supplementary Figure S1 [file bty374_figure_s1.pdf]

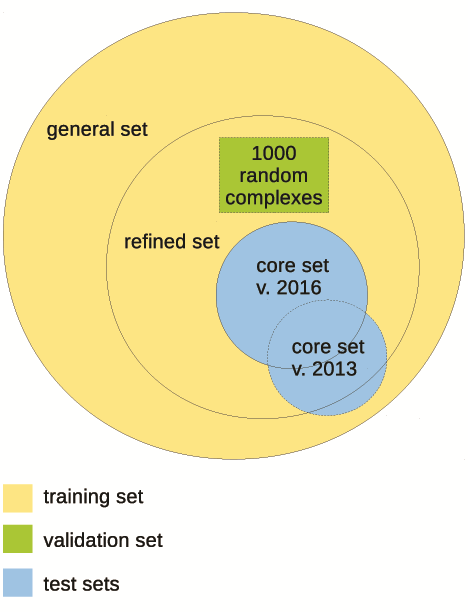

Supplement: Supplementary Figure S2 [file bty374_figure_s2.png]

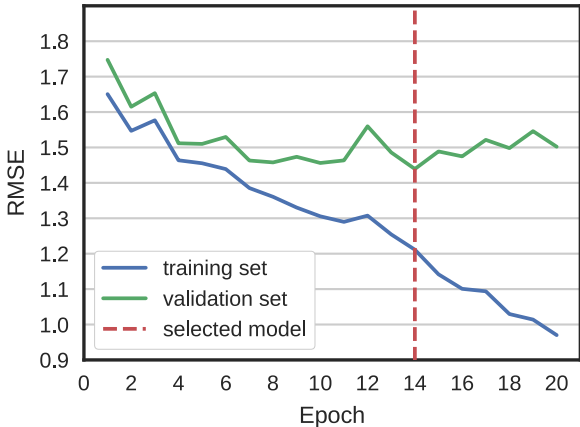

Supplement: Supplementary Figure S3 [file bty374_figure_s3.pdf]

PDB ID

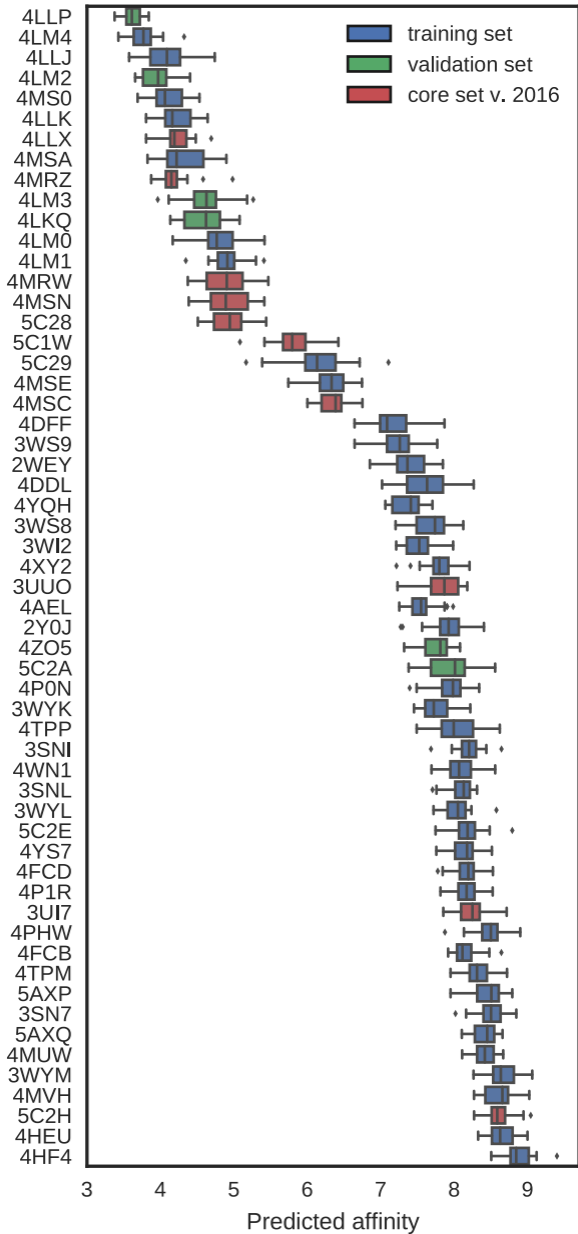

Supplement: Supplementary Figure S4 [file bty374_figure_s4.pdf]
